# A Pilot Randomized Control Trial With the Probiotic Strain *Lactobacillus rhamnosus* GG (LGG) in ADHD: Children and Adolescents Report Better Health-Related Quality of Life

**DOI:** 10.3389/fpsyt.2020.00181

**Published:** 2020-03-17

**Authors:** Hojka Gregoric Kumperscak, Alja Gricar, Ina Ülen, Dusanka Micetic-Turk

**Affiliations:** ^1^Pediatric Clinic, University Medical Center Maribor, Maribor, Slovenia; ^2^Faculty of Medicine, University of Maribor, Maribor, Slovenia; ^3^Community Health Center Dr. Adolf Drolc, Maribor, Slovenia

**Keywords:** attention-deficit/hyperactivity disorder, ADHD, *Lactobacillus rhamnosus* GG, LGG, probiotics, children, adolescents, health-related quality of life

## Abstract

**Objectives:** This double-blind pilot randomized placebo-controlled trial examined the possible effect of the probiotic strain *Lactobacillus rhamnosus* GG ATCC53103 (LGG) on symptoms of attention-deficit/hyperactivity disorder (ADHD), health-related quality of life (QoL), and serum levels of cytokines in children and adolescents with ADHD.

**Methods:** This trial evaluated 32 drug-naive children and adolescents aged between four and 17 years with a diagnosis of ADHD. The study subjects were randomly assigned to either the group that received LGG or the group that received the placebo. Assessments, comprising *the ADHD Parent-Report Rating Scale-IV: Home Version*; *the Child Self-Report and Parent Proxy-Report of the Pediatric Quality of Life Inventory*^TM^ *(PedsQL*^TM^*) 4.0 Generic Core Scale; the Parent Form (CBCL/6-18) and the Teacher Report Form (TRF) of the Child Behavior Checklist (CBCL) for ages 6–18 of the Achenbach System of Empirically Based Assessment (ASEBA)*; and the serum cytokines; were compared between the groups at the baseline and after 3 months.

**Results:** Thirty-five participants were randomized, with 32 completing the study (91.4% retention). There was a significant improvement in the PedsQL Child Self-Report Total Score after 3 months of treatment in the probiotic (*p* = 0.021, d = 0.53), whereas there was no significant improvement in the placebo group (*p* = 0.563, d = 0.04). The results of psychometric parameters assessed by parents and teachers were not so straightforward. There were statistically significant differences in the levels of serum cytokines between the groups after the 3-month treatment period: IL-6 in both the probiotic (*p* = 0.004, d = 0.73) and the placebo groups (*p* = 0.035, d = 0.94); IL-10 (*p* = 0.035, d = 0.6); IL-12 p70 (*p* = 0.025, d = 0.89); and TNF-α (*p* = 0.046, d = 0.64) in the probiotic group only.

**Conclusions:** Children and adolescents with ADHD who received LGG supplementation reported better health-related QoL compared to their peers who received the placebo. This suggests that LGG supplementation could be beneficial. But results with psychometric tests conducted by parents and teachers as well as differences in the levels of inflammatory cytokines were ambiguous. Based on these results, we propose some study modifications: a longer observation period (6–12 months); inclusion of more children's self-report assessments; recruitment of non-drug naive patients and the possible omission of serum cytokines measurements.

**Clinical Trial Registration:** Medical Ethics Committee (UKC-MB-KME-19-06/16).

## Introduction

Attention-deficit/hyperactivity disorder (ADHD) is nowadays the most prevalent neurodevelopmental disorder with an estimated worldwide-pooled prevalence of 5.29% among people up to and including 18 years of age (1). According to the Diagnostic and Statistical Manual of Mental Disorders-Fifth Edition (DSM-5), the diagnosis of ADHD is based on clinical symptoms (2). The recommended treatment for ADHD is multimodal, covering every aspect of children's psychosocial environment (3, 4).

Pharmacological treatments are efficacious and are widely used, but they may have several limitations (5). For many reasons, but mostly because of side effects, families choose not to use pharmacotherapy and non-adherence to therapy is rather high. Therefore, more research is needed to explain the etiology of ADHD and the possible benefits of non-pharmacological therapies (6, 7). A variety of non-pharmacological interventions are available, but their efficacy remains uncertain (5). There has been a growing interest in dietary interventions (5). According to a systematic review and meta-analyses of RCT, free fatty acid supplementation produced small but significant reductions in ADHD symptoms even with probably blinded assessments, although the clinical significance of these effects remains to be determined (5). Artificial food color exclusion produced larger effects, although this was often in individuals selected because of food sensitivities (5).

Genetics has been described as the most important etiological factor in ADHD, but the contribution of environmental factors has been estimated to be 20–30% (1, 8). The search for possible etiological factors has been expanded in recent years to include the gut microbiota (7, 9). According to experimental evidence from animal models, gut microbiota is involved in brain development and function and may play a crucial role in neurotransmission and neuronal plasticity, as explained in the gut-brain axis (7, 10–15). The reverse is also true, with intestinal dysbiosis being linked to various behavioral features in psychiatric disorders (15). A study by Borre demonstrated that early-life perturbations of the developing gut microbiota, which is very vulnerable because of its high instability and immaturity, can influence neurodevelopment and potentially lead to adverse mental health outcomes later in life (16). There is evidence from animal studies that the gut microbiota might also be a therapeutic target in ADHD, mediated by the deregulation of neurotransmitters (17, 18).

Proposed pathways by which the gut microbiota may modulate brain development, function, and behavior, are immune (cytokines), metabolic (short-chain fatty acids), endocrine (cortisol), and neuronal (the vagus nerve and the enteric nervous system) pathways (19). For example, host (human) stress hormones such as norepinephrine might influence bacterial gene expression or signaling between bacteria and thus change the microbial composition and activity (20). Certain microbiota can produce neuroactive compounds such as neurotransmitters (7). *Lactobacillus* spp. and *Bifidobacterium* spp. can produce GABA and *Lactobacillus* spp. also produces acetylcholine, etc. (21, 22).

Several studies have proposed an immune pathway by which gut microbiota could modulate brain development in ADHD (23–25) and these are supported by findings of increased serum levels of proinflammatory cytokines such as IFN-γ and IL-16 in ADHD patients (6). Blood test parameters, for which values have been found to be significantly different in children with ADHD, are the biochemical markers CRP, B-12, folate, iron, ferritin, transferrin, and the cytokines IL-6, IL-10, and IL-16 (26–29). Blood parameters that have been found to be changed by LGG with statistical significance are TNF-α, IL-1β, IL-2, IL-4, IL-6, IL-8, IL-10, IL-12, IL-17, and IL-18. IL-4 and IL-10 are anti-inflammatory, while the others are proinflammatory cytokines (30–38).

A study by Isolauri on the modulation of the maturing gut barrier showed that LGG stabilizes the gut permeability barrier by its effects on tight junctions, mucin production and antigen-specific immunoglobulin A production (39). Experimental studies by Bravo and Enticott have demonstrated that LGG, via the vagus nerve, regulates emotional behavior and the central GABAergic system, which is also associated with neuropsychiatric disorders (13, 40). Of note is a recent study in healthy women that demonstrated that the consumption of a mixture of probiotic bacteria had significant effects on brain regions that control the central processing of emotion and sensation (41).

In a recent human study by Pärtty, preliminary results demonstrated that specific probiotics may reduce the risk of the development of ADHD and other neurodevelopmental disorders (9). Pärtty's data showed that the administration of LGG during the first 6 months of a child's life may reduce the risk of ADHD and ASD later in childhood by mechanisms not limited to the composition of the gut microbiota. They demonstrated a possible preventive risk-reducing effect of the probiotic LGG on the later development of ADHD and Asperger syndrome. This is one of the few human studies on the possible effect of probiotics on ADHD. Although the results are encouraging, they should be interpreted with caution and need to be confirmed by further studies (7, 9).

The primary objective of our pilot study was to investigate the possible effect of LGG on ADHD symptomatology, health-related QoL and serum levels of pro- and anti-inflammatory cytokines in drug-naïve children and adolescents with ADHD. The secondary objective was to check if the study design is solid enough or whether some changes are needed, for example, whether the duration of the study is long enough to observe the changes in the chosen parameters and to determine the main obstacles to patient recruitment.

## Methods

### Experimental Design

The present study was a countrywide, double-blind, randomized, parallel-group prospective placebo-controlled trial conducted from January 2018 to April 2018 on child and adolescent outpatients with ADHD. The protocol, which is consistent with the Declaration of Helsinki and its successive revisions, was approved by the Medical Ethics Committee (UKC-MB-KME-19-06**/**16) before implementation. After a complete description and comprehensive explanation of the procedures, the purpose of the study, and the reassurance of confidentiality, written informed consent was obtained from the patients or their legal guardians. Each patient and their guardian were told that they were free to withdraw from the trial without any negative effect on their treatment. All the participants or their parents accordingly provided written informed consent.

At the baseline visit, participants were blindly randomized into two groups (placebo and probiotic groups). They were instructed to take one capsule once daily for a period of 3 months. The shape, color, and weight of capsules with the placebo and the probiotic were identical. Probiotic capsules contained the probiotic strain *Lactobacillus rhamnosus* GG (at least 10^10^ CFU) and the excipients hydroxypropyl methylcellulose (E464), maltodextrins and the coloring titanium dioxide (E171). The ingredients in the placebo were the same as in the probiotic capsule except for the probiotic strain. Participants were assessed twice—at the baseline visit (t_0_) and after 3 months (t_1_).

### Subjects

ADHD patients aged between four and 17 years were invited to participate in the study. They were recruited from child and adolescent psychiatry outpatient clinics in Slovenia. The clinical diagnosis of ADHD according to DSM-5 was made prior to the study by the participant's child and adolescent psychiatrist. Exclusion criteria were: age <4 years or more than 17 years; taking pharmacotherapy for ADHD; or being diagnosed with HIV/AIDS, Crohn's disease, ulcerative colitis or cancer; or receiving chemotherapy.

### Measurements

#### ADHD Symptomatology and Health-Related QoL

The following ratings were performed at t_0_ and t_1_: the ADHD Parent-Report Rating Scale-IV: Home Version; the Child Self-Report and Parent Proxy-Report of the Pediatric Quality of Life Inventory^TM^ (PedsQL^TM^) 4.0 Generic Core Scale; and the Parent Form (CBCL/6-18) and the Teacher Report Form (TRF) of the Child Behavior Checklist (CBCL) for ages 6–18 of the Achenbach System of Empirically Based Assessment (ASEBA).

*The ADHD Rating Scale-IV: Home Version* is a behavioral questionnaire based on the diagnostic criteria for ADHD as described in the fourth edition of the Diagnostic and Statistical Manual of Mental Disorders (DSM-IV). It obtains parent ratings regarding the frequency of each ADHD symptom (42).

The *PedsQL Measurement Model* is a modular approach to measuring health-related quality of life (QoL) in healthy children and adolescents and those with acute and chronic health conditions. The 23-item *PedsQL*^TM^ *Generic Core Scales* are multidimensional *Child Self-Report* and *Parent Proxy-Report* scales designed to measure the core dimensions of health as delineated by the World Health Organization, as well as role (school) functioning. The scales assess physical, emotional, social, and school functioning (43–45). We used raw scores response choices, where low scores indicate a better health-related quality of life (QoL).

The *Parent Form (CBCL/6-18)* and the *Teacher Report Form (TRF)* of the *Child Behavior Checklist (CBCL)* for ages 6–18 of the Achenbach System of Empirically Based Assessment (ASEBA) are widely used forms identifying problems in children (46, 47).

#### Pro- and Anti-inflammatory Cytokines

Serum was isolated from blood collected from patients at the baseline and after 3 months. The serum was simultaneously analyzed for the following cytokines: IL-1B, IL-2R, IL-4, IL-6, IL-8, IL-10, IL-12 p70, IL-17, IL-18, and TNF-α. The serum samples for determination of cytokines were measured by Luminex Multiplex Technology using the RD-LXSAH-09 Luminex Human Assay. If the values of results were below the limit of detection (LOD), LOD was used as the result for analysis.

#### Safety

The participants were asked to immediately inform the research team of any unexpected symptom or complaint during the study period. They were systematically asked for any side effects throughout the study using a daily checklist. Supplement adherence was measured by a review of daily supplement intake reports.

#### Statistical Analysis

Non-parametric tests were used due to the small sample size. The Wilcoxon signed-rank test was used to calculate the differences between baseline and follow-up measurements. The Mann-Whitney *U*-test was used to calculate baseline differences and follow-up change between the groups. The Mann-Whitney *U*-test and Fisher's exact test were used to calculate baseline differences between the groups in age and gender, respectively. Statistical analyses were made using IBM SPSS 25 software (IBM Corp., Armonk, NY). A probability level of *p* < 0.05 was considered statistically significant. Results are presented as mean (M) and standard deviation (SD).

## Results

**Figure 1:**
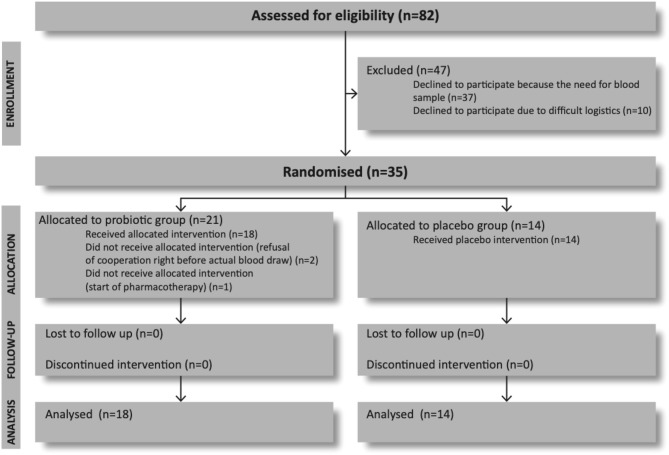
Flow diagram of the progress through the phases of a parallel randomized trial of probiotic and placebo group (according to CONSORT 2010 Flow Diagram).

Out of 82 invited patients who met the study criteria, 37 (45.1%) refused to take part because of the need for blood sample examination (Figure 1). Ten (12.2%) further participants dropped out because of distance and transportation. Thirty-five participants (42.7%) entered the trial and were blindly randomized to receive either the probiotic or the placebo. Further three patients who were randomized in probiotic group were excluded from the study before collection of their baseline data. One was excluded because of starting pharmacotherapy which was an exclusion criterion, and the other two because they refused to give blood sample on the baseline visit.

Thirty-two participants completed the trial, 18 in the probiotic and 14 in the placebo group. Altogether 39 (47.6%) participants refused intervention because of need for blood sample. There were no drops out in follow-up period.

In the probiotic group (*N* = 18), there were 12 males and 6 females with median age 11.0 years (M 11.4 ± 3.2). In the placebo group (*N* = 14), there were 11 males and 3 females with median age of 13.0 years (M 12.5 ± 2.3). Statistical analyses did not show a significant difference in any baseline characteristics between the groups, including gender (*p* = 0.694) and age (*p* = 0.059), nor in any other characteristic (Table 1). Change from baseline to follow-up between the groups showed significant improvement on PedQL Child Self-Report (*p* = 0.044) but in no other characteristic (Table 2).

**Table 1:** Baseline clinical parameters of patients in probiotic and placebo group.

	**Probiotic group (*N* = 18)**	**Placebo group (*N* = 14)**	***p***
Race—Caucasian *N (%)*	18 (100)	14 (100)	1.000^*^
Gender *N (%)*			0.694^*^
Female	6 (33.3)	3 (21.4)	
Male	12 (66.6)	11 (78.6)	
Age (in years) *M (SD)*	11.4 (3.2)	12.5 (2.3)	0.059^#^
**Proinflammatory cytokines** ***M (SD)***			
IL-1β (pg/mL)	29.3 (30.4)	27.1 (18.2)	0.759^#^
IL-2R (pg/mL)	35.9 (14.3)	208.2 (661.8)	0.828^#^
IL-6 (pg/mL)	16.3 (2.0)	17.3 (2.6)	0.142^#^
IL-8 (pg/mL)	29.4 (14.0)	39.3 (29.8)	0.736^#^
IL-12 p70 (pg/mL)	225.8 (147.2)	254.9 (172.0)	0.627^#^
IL-17 (pg/mL)	27.5 (14.6)	19.5 (13.9)	0.183^#^
IL-18 (pg/mL)	157.7 (72.2)	170.8 (70.6)	0.842^#^
TNF-α (pg/mL)	31.9 (1.6)	32.5 (1.6)	0.272^#^
**Anti-inflammatory cytokines M (SD)**			
IL-4 (pg/mL)	15.7 (0.0)	19.6 (14.7)	0.257^#^
IL-10 (pg/mL)	13.9 (0.9)	14.1 (1.4)	0.838^#^
**Health-related quality of life and ADHD symptomatology M (SD)**			
PedsQL Child Self-Report Total Score	24.8 (11.0)	25.9 (17.4)	0.747^#^
PedsQL Parent Proxy-Report Total Score	28.8 (11.1)	36.6 (16.8)	0.160^#^
ADHD Rating Scale	33.7 (7.9)	35.1 (10.5)	0.775^#^
Child Behavior Checklist Parent Form Total Score	50.8 (18.3)	49.9 (22.5)	0.525^#^
Child Behavior Checklist Teacher Report Form Total Score	44.4 (25.3)	54.4 (17.3)	0.284^#^

* p < 0.05; *Fisher's exact test; ^#^Mann-Whitney U-test (baseline comparison between the groups).

*N, numerus; M, mean; SD, standard deviation*.

**Table 2:** Results of the Wilcoxon signed-rank tests for health-related quality of life and ADHD symptomatology, comparing the probiotic and placebo group scores.

	**Probiotic group (*****N*** **=** **18)**					**Placebo group (*****N*** **=** **14)**					***p^***$***^***
	**Baseline M (SD)**	**Follow-upM (SD)**	**Δ** **M (SD)**	***p^**#**^***	***d***	**BaselineM (SD)**	**Follow-up M (SD)**	**ΔM (SD)**	***p^**#**^***	***d***	
PedsQL Child Self-Report Total Score	24.8 (11.0)	19.8 (8.1)	−5.1 (9.0)	0.021^*^	0.53	25.9 (17.4)	25.2 (13.8)	−0.6 (8.1)	0.563	0.04	0.044
PedsQL Parent Proxy-Report Total Score	28.8 (11.1)	22.7 (10.4)	−6.2 (13.0)	0.078	0.57	36.6 (16.8)	26.8 (12.3)	−9.7 (10.1)	0.021^*^	0.67	0.598
ADHD Rating Scale	33.7 (7.9)	26.4 (9.3)	−7.2 (8.7)	0.008^*^	0.84	35.1 (10.5)	27.7 (16.3)	−7.4 (11.2)	0.021^*^	0.54	0.952
Child Behavior Checklist Parent Form Total Score	50.8 (18.3)	37.0 (14.6)	−13.8 (20.6)	0.003^*^	0.84	49.9 (22.5)	34.8 (14.6)	−15.1 (16.2)	0.008^*^	0.80	0.410
Child Behavior Checklist Teacher Report Form Total Score	44.4 (25.3)	35.6 (20.0)	−8.9 (24.2)	0.185	0.39	54.4 (17.3)	55.3 (25.2)	0.9 (15.5)	0.889	0.04	0.296

* p < 0.05;

# *Wilcoxon signed-rank test (follow-up vs. baseline comparison for each group separately)*.

Δ follow-up minus baseline;

$ *Mann-Whitney U-test (Δ comparison between the groups)*.

*N, numerus; M, mean; SD, standard deviation; d, The Cohen's d effect size*.

### ADHD Symptomatology and Health-Related QoL

#### Self-Report

Results for ADHD symptomatology and health-related QoL are presented in Table 2. There was a statistically significant improvement in the PedsQL Child Self-Report Total Score in the probiotic group (*p* = 0.021, d = 0.53), but not in the placebo group (*p* = 0.563, d = 0.04). Mann-Whitney U test showed significant difference between groups in the change from baseline to follow-up in this characteristic (*p* = 0.044).

#### Parent-Report

On the PedsQL Parent Proxy-Report, parents reported a statistically significant improvement in the Total Scale Score in the placebo group (*p* = 0.021, d = 0.67) but a non-significant improvement in the probiotic group (*p* = 0.078, d = 0.57). Statistically significant improvement on the ADHD Rating Scale score was observed by parents in both groups, the probiotic group (*p* = 0.008, d = 0.84) and the placebo group (*p* = 0.021, d = 0.54), respectively. There was a significant improvement in overall behavior observed by parents with the CBCL Parent Form in both groups, the probiotic group (*p* = 0.003, d = 0.84) and the placebo group (*p* = 0.008, d = 0.80), respectively.

#### Teacher-Report

Teachers did not report significant improvement in overall behavior with the CBCL Teacher Report Form Total Score, the probiotic group (*p* = 0.185, d = 0.39) and the placebo group (*p* = 0.889, d = 0.04), respectively.

#### Cytokines Levels

Results for serum cytokine levels are presented in Table 3. Among proinflammatory cytokines, there was a statistically significant decreasing in the probiotic group regarding serum levels of IL-6 (*p* = 0.004, d = 0.73), IL-12 p70 (*p* = 0.025, d = 0.89), and TNF-α (*p* = 0.046, d = 0.64), of which IL-12 p70 and TNF-α were significantly reduced only in the probiotic group. In the placebo group, a reduction was observed in the IL-6 level (*p* = 0.035, d = 0.94). No other proinflammatory cytokines showed a statistically significant change in any of the groups in the 3 months of treatment. Among the anti-inflammatory cytokines, serum levels of IL-10 were statistically significantly lower (*p* = 0.035, d = 0.60) in the probiotic group after 3 months of treatment, while there was no significant change in the placebo group. There was no significant change after 3 months in IL-4 serum levels in either of the groups. Mann-Whitney U test showed no difference between groups in the change from baseline to follow-up in serum level of any cytokine.

**Table 3:** Results of the Wilcoxon signed-rank tests for inflammatory cytokines, comparing probiotic and placebo group scores.

	**Probiotic group (*****N*** **=** **18)**					**Placebo group (*****N*** **=** **14)**					***p^***$***^***
	**Baseline M (SD)**	**Follow-upM (SD)**	**Δ** **M (SD)**	***p^**#**^***	***d***	**BaselineM (SD)**	**Follow-up M (SD)**	**ΔM (SD)**	***p^**#**^***	***d***	
**Proinflammatory cytokines**											
IL-1β (pg/mL)	29.3 (30.4)	17.9 (0.0)	−11.4 (30.4)	0.102	0.53	27.1 (18.2)	23.9 (15.2)	−3.2 (20.3)	0.414	0.19	0.558
IL-2R (pg/mL)	35.9 (14.3)	31.3 (0.6)	−4.7 (14.3)	0.109	0.46	208.2 (661.8)	31.3 (0.7)	−176.9 (661.8)	0.414	0.38	0.546
IL-6 (pg/mL)	16.3 (2.0)	14.6 (2.6)	−1.7 (2.8)	0.004^*^	0.73	17.3 (2.6)	14.0 (4.1)	−3.3 (6.4)	0.035^*^	0.94	0.872
IL-8 (pg/mL)	29.4 (14.0)	33.5 (17.6)	4.9 (21.9)	0.149	0.26	39.3 (29.8)	38.5 (29.2)	−0.8 (46.8)	0.551	0.03	0.953
IL-12 p70 (pg/mL)	225.8 (147.2)	133.6 (0.0)	−92.2 (147.2)	0.025^*^	0.89	254.9 (172.0)	156.0 (83.8)	−98.9 (165.3)	0.059	0.73	0.940
IL-17 (pg/mL)	27.5 (14.6)	20.3 (13.0)	−7.3 (19.4)	0.083	0.52	19.5 (13.9)	20.5 (13.2)	1.1 (15.0)	1.000	0.08	0.182
IL-18 (pg/mL)	157.7 (72.2)	161.7 (74.7)	4.1 (68.8)	0.638	0.05	170.8 (70.6)	136.7 (56.4)	−34.1 (75.1)	0.173	0.53	0.340
TNF-α (pg/mL)	31.9 (1.6)	27.8 (8.7)	−4.2 (8.2)	0.046^*^	0.64	32.5 (1.6)	28.4 (8.3)	−4.0 (6.8)	0.103	0.69	0.796
**Anti-inflammatory cytokines**											
IL-4 (pg/mL)	15.7 (0.0)	18.8 (12.9)	3.1 (12.9)	0.317	0.33	19.6 (14.7)	15.7 (0.0)	−3.9 (14.7)	0.317	0.38	0.161
IL-10 (pg/mL)	13.9 (0.9)	13.4 (0.6)	−0.5 (0.8)	0.035^*^	0.60	14.1 (1.4)	13.4 (0.6)	−0.6 (1.6)	0.066	0.58	0.691

* p < 0.05;

# *Wilcoxon signed-rank test (follow-up vs. baseline comparison for each group separately)*.

Δ follow-up minus baseline;

$ *Mann-Whitney U-test (Δ comparison between the groups)*.

*N, numerus; M, mean; SD, standard deviation; d, The Cohen's d effect size*.

#### Supplement Adherence and Adverse Events

Supplement adherence was measured by a review of daily supplement intake reports and was 100%. No adverse events were reported by parents in either the probiotic or the placebo group.

## Discussion

The most interesting finding of the present study was a significant improvement in the health-related QoL the PedsQL Child Self-Report only in the probiotic group reflected by a medium effect size (48). These results could implicate that children and adolescents who took LGG supplementation felt better and reported better physical, emotional, social, and school functioning. They had better health-related QoL compared to their peers who were not taking LGG supplementation. As noted by Coghill and Hodgkins (49), most studies on ADHD and QoL only obtained assessments from the child's parents and therapists, although the child's view about his own symptoms and QoL is of vital importance.

On the other hand, the results were not so straightforward when parents assessed their children.

A significant reduction in ADHD symptomatology was observed by parents on the ADHD Rating Scale with large effect size in probiotic and medium effect size in placebo group. On the CBCL Parent Form, there was also a significant improvement in overall behavior observed by parents in both groups with large effect size in probiotic as well as in placebo group. On the PedsQL Parent Proxy-Report, the parents reported a significant improvement in health-related QoL only in the placebo group (medium effect size). This could be interpreted in several ways: enrollment in the study changed the parent-child relationship; parents gave their children more positive attention regardless of whether they received LGG or the placebo; parents from both groups observed their children more carefully after their enrollment in the study; and they probably also spent more time together. These factors could result in better parent-child attachment and reflect positively on how parents scored their children, regardless of whether they received LGG or the placebo.

Teachers did not report significant improvement in either of the groups over the study period in overall behavior assessed by the CBCL Teacher Report Form. A possible explanation for these results could be that teachers were less motivated to participate in the study than parents and less diligent in completing the report forms.

LGG intervention showed a significant reduction in the production of the proinflammatory IL-12 p70 (large effect size) and TNF-α cytokines (medium effect size) in the probiotic group only, but also a significant reduction in the proinflammatory IL-6 in both groups with large effect size in placebo and medium in probiotic group. The results also showed that LGG significantly decreased the anti-inflammatory IL-10 cytokines (medium effect size) only in the probiotic group. It is challenging to interpret these ambiguous results. It is possible that the duration of the study was too short to result in more straightforward changes in the pro- and anti-inflammatory cytokines. This is one of the reasons that we propose to change the study design to a longer trial period of 6–12 months.

The need for blood sample proved to be the main reason of refusal of participation in the study. Nearly half of the invited patients declined to participate because of this in different stages of study. But there were no dropouts in the follow-up period. The feasibility of the study was indicated by excellent compliance and tolerance of both the probiotic and the placebo group. No adverse events were reported by parents in either group.

The strength of the present study was its double-blind randomized placebo-controlled design in drug-naïve patients, which enables clear data interpretation. This is, to our knowledge, the first study observing the possible LGG effects on children and adolescents with ADHD. A very important fact is that ADHD symptomatology was assessed not only by parents but also by teachers and the children themselves.

The limitation of the study was its small sample size, which is normal for pilot studies. This study involved 32 participants, which means differences in the results of the questionnaires between t_0_ and t_1_ had to be much greater to achieve statistical significance than would be necessary for a study with a larger number of participants. Small samples can, in fact, prevent the extrapolation of data (50, 51). Despite the small sample size, the proven calculated statistically significant values should be considered to be important.

Results of the present pilot study suggests that larger RCT is warranted, but we propose some changes in the study design. Firstly, the experimental period should be longer (6–12 months). This may enable us to detect more significant changes in both psychometric and serum parameters. Secondly, it would be important to include more children's observational assessments since the children reported unambiguous results and studies that include the child's view about his own symptoms and QoL are rare. Thirdly, the study population should include patients on stable pharmacotherapy. Most of the ADHD patients in outpatient clinics do receive medication. Because of the exclusion criteria that patients must not be taking ADHD medication, most ADHD patients could not be enrolled in the study. Therefore, including non-drug-naïve patients will enable a much larger sample size.

It is questionable whether the pro- and anti-inflammatory cytokines should be included in a new study design. The results of the pilot study on these are hard to interpret. The tests are expensive and they represent the major costs of the study. We could have recruited many more patients in the pilot study if blood samples for cytokines testing had not been needed. However, the results of the pro- and anti-inflammatory cytokines could be significantly changed over a longer period and with a larger sample size.

## Conclusions

In the 3-month-double-blind RCT, children and adolescents with ADHD who received LGG supplementation reported better physical, emotional, social, and school functioning. They had better health-related QoL compared to their peers in the placebo group. This could suggest that LGG supplementation could be beneficial regarding the health-related QoL of children with ADHD.

The results of the study are quite ambiguous when parents and teachers assess ADHD symptomatology and health-related QoL during the study. The results on pro- and anti-inflammatory cytokines are not straightforward and are hard to interpret. Based on these pilot study results we suggest that larger RCT is warranted but with some changes in the study design: longer duration of the study, the inclusion of more children's self-report assessments, and the recruitment of non-drug-naïve patients. There are pros and cons regarding the inclusion of cytokine tests.

## Data Availability Statement

All datasets generated for this study are included in the article/supplementary material.

## Ethics Statement

The studies involving human participants were reviewed and approved by the Medical Ethics Committee of University Clinical center Maribor (UKC-MB-KME-19-06/16). Written informed consent to participate in this study was provided by the participants' legal guardian/next of kin.

## Author Contributions

HK: study design, data interpretation, article review and revisions, and final approval of the article. AG and IÜ: patient enrollment, collection and assembly of data, data interpretation, and final approval of the article. DM-T: study design, article review and revisions, and final approval of the article.

### Conflict of Interest

HK receives or has received research support from the Slovenian Research Agency, been an advisory board member and served as a speaker for Pliva, Krka, Janssen Cilag, and Eli Lilly. The remaining authors declare that the research was conducted in the absence of any commercial or financial relationships that could be construed as a potential conflict of interest.

## References

[1] Polanczyk G de Lima MS Horta BL Biederman J Rohde LA. The worldwide prevalence of ADHD: a systematic review and metaregression analysis. Am J Psychiatry. (2007) 164:942–8. 10.1176/ajp.2007.164.6.942 17541055

[2] American Psychiatric Association Diagnostic and Statistical Manual of Mental Disorders. 5th ed. Arlington, VA: American Psychiatric Publishing (2013).

[3] Canadian Attention Deficit Hyperactivity Disorder Resource Alliance Canadian ADHD Practice Guidelines (CAP-Guidelines). 3rd ed. Canadian Attention Deficit Hyperactivity Disorder Resource Alliance (Toronto, ON: CADDRA) (2011).

[4] Taylor E Döpfner M Sergeant J Asherson P Banaschewski T Buitelaar J. European clinical guidelines for hyperkinetic disorder - first upgrade. Eur Child Adolesc Psychiatry. (2004) 13(Suppl.):17–30. 10.1007/s00787-004-1002-x 15322953

[5] Sonuga-Barke EJS Brandeis D Cortese S Daley D Ferrin M Holtmann M. Nonpharmacological interventions for ADHD: systematic review and meta-analyses of randomized controlled trials of dietary and psychological treatments. Am J Psychiatry. (2013) 170:275–89. 10.1176/appi.ajp.2012.12070991 23360949

[6] Verlaet AA Noriega DB Hermans N Savelkoul HF. Nutrition, immunological mechanisms and dietary immunomodulation in ADHD. Eur Child Adolesc Psychiatry. (2014) 23:519–29. 10.1007/s00787-014-0522-2 24493267

[7] Cenit MC Nuevo IC Codoñer-Franch P Dinan TG Sanz Y. Gut microbiota and attention deficit hyperactivity disorder: new perspectives for a challenging condition. Eur Child Adolesc Psychiatry. (2017) 26:1081–92. 10.1007/s00787-017-0969-z 28289903

[8] Van Loo KM Martens GJ. Genetic and environmental factors in complex neurodevelopmental disorders. Curr Genomics. (2007) 8:429–44. 10.2174/138920207783591717 19412416 PMC2647153

[9] Pärtty A Kalliomäki M Wacklin P Salminen S Isolauri. A possible link between early probiotic intervention and the risk of neuropsychiatric disorders later in childhood: a randomized trial. Pediatr Res. (2015) 77:823–8. 10.1038/pr.2015.51 25760553

[10] Bercik P Verdu EF Foster JA Macri J Potter M Huang X. Chronic gastrointestinal inflammation induces anxiety-like behavior and alters central nervous system biochemistry in mice. Gastroenterology. (2010) 139:2102–12. 10.1053/j.gastro.2010.06.063 20600016

[11] Bercik P Park AJ Sinclair D Khoshdel A Lu J Huang X. The anxiolytic effect of bifidobacterium longum NCC3001 involves vagal pathways for gut-brain communication. Neurogastroenterol Motil. (2011) 23:1132–9. 10.1111/j.1365-2982.2011.01796.x 21988661 PMC3413724

[12] Neufeld KM Kang N Bienenstock J Foster JA. Reduced anxiety-like behavior and central neurochemical change in germ-free mice. Neurogastroenterol Motil. (2011) 23:255–64. 10.1111/j.1365-2982.2010.01620.x 21054680

[13] Bravo JA Forsythe P Chew MV Escaravage E Savignac HM Dinan TG. Ingestion of Lactobacillus strain regulates emotional behavior and central GABA receptor expression in a mouse via the vagus nerve. Proc Natl Acad Sci USA. (2011) 108:16050–5. 10.1073/pnas.1102999108 21876150 PMC3179073

[14] Messaoudi M Lalonde R Violle N Javelot H Desor D Nejdi A. Assessment of psychotropic-like properties of a probiotic formulation (*Lactobacillus helveticus* R0052 and *Bifidobacterium longum* R0175) in rats and human subjects. Br J Nutr. (2010) 105:755–64. 10.1017/S0007114510004319 20974015

[15] Dinan TG Cryan JF The impact of gut microbiota on brain and behavior: implications for psychiatry. Curr Opin Clin Nutr Metab Care. (2015) 18:552–8. 10.1097/MCO.0000000000000221 26372511

[16] Borre YE O'Keeffe GW Clarke G Stanton C Dinan TG Cryan JF. Microbiota and neurodevelopmental windows: implications for brain disorders. Trends Mol Med. (2014) 20:509–18. 10.1016/j.molmed.2014.05.002 24956966

[17] Cryan JF Dinan TG. More than a gut feeling: the microbiota regulates neurodevelopment and behavior. Neuropsychopharmacology. (2014) 40:241–2. 10.1038/npp.2014.224 25482171 PMC4262908

[18] Luczynski P McVey Neufeld KA Oriach CS Clarke G Dinan TG Cryan JF. Growing up in a bubble: using germ-free animals to assess the influence of the gut microbiota on brain and behavior. Int J Neuropsychopharmacol. (2016) 19:1–17. 10.1093/ijnp/pyw020 26912607 PMC5006193

[19] Rogers GB Keating DJ Young RL Wong ML Licinio J Wesselingh S. From gut dysbiosis to altered brain function and mental illness: mechanisms and pathways. Mol Psychiatry. (2016) 21:738–48. 10.1038/mp.2016.50 27090305 PMC4879184

[20] Collins SM Bercik P The relationship between intestinal microbiota and the central nervous system in normal gastrointestinal function and disease. Gastroenterology. (2009) 6:2003–14. 10.1053/j.gastro.2009.01.075 19457424

[21] Barrett E Ross RP O'Toole PW Fitzgerald GF Stanton C. γ-Aminobutyric acid production by culturable bacteria from the human intestine. J Appl Microbiol. (2012) 113:411–7. 10.1111/j.1365-2672.2012.05344.x 22612585

[22] Dinan TG Stanton C Cryan JF. Psychobiotics: a novel class of psychotropic. Biol Psychiatry. (2013) 74:720–6. 10.1016/j.biopsych.2013.05.001 23759244

[23] Yamashiro Y. Gut microbiota in health and disease. Ann Nutr Metab. (2017) 71:242–6. 10.1159/000481627 29136611

[24] Chung H Kasper DL. Microbiota-stimulated immune mechanisms to maintain gut homeostasis. Curr Opin Immunol. (2010) 22:455–46. 10.1016/j.coi.2010.06.008 20656465

[25] Atarashi K Honda K. Microbiota in autoimmunity and tolerance. Curr Opin Immunol. (2011) 23:761–8. 10.1016/j.coi.2011.11.002 22115876

[26] Faraone SV Bonvicini C Scassellati C. Biomarkers in the diagnosis of ADHD – promising directions. Curr Psychiatry Rep. (2014) 16:497. 10.1007/s11920-014-0497-1 25298126

[27] Donfrancesco R Nativio P Di Benedetto A Villa MP Andriola E Melegari MG Anti-Yo antibodies in children with ADHD: first results about serum cytokines. J Atten Disord. (2016) 20:1–6. 10.1177/1087054716643387 27095560

[28] Hariri M Djazayery A Djalali M Saedisomeolia A Rahimi A Abdolahian E. Effect of n-3 supplementation on hyperactivity, oxidative stress and inflammatory mediators in children with attention-deficit-hyperactivity disorder. Malays J Nutr. (2012) 18:329–35. 24568073

[29] Garipardic M Dogan M Bala KA Mutluer T Kaba S Aslan O. Association of attention deficit hyperactivity disorder and autism spectrum disorders with mean platelet volume and vitamin D. Med Sci Monit. (2017) 23:1378–84. 10.12659/msm.899976 28319054 PMC5370427

[30] Fang S Zhang Y Zhang Y Zhu X Yie B *Lactobacillus rhamnosus* GG improves symptoms and its mechanism in mice with ovalbumin-induced food allergy. Xi Bao Yu Fen Zi Mian Yi Xue Za Zhi. (2017) 33:597–600. 28502295

[31] Miyazawa K Yoda K Kawase M Harata G He F. Influence of orally administered Lactobacillus GG on respiratory immune response in a murine model of diet-induced obesity. Microbiol Immunol. (2015) 59:99–103. 10.1111/1348-0421.12226 25643737

[32] Wang H Gao K Wen K Allen IC Li G Zhang W. *Lactobacillus rhamnosus* GG modulates innate signaling pathway and cytokine responses to rotavirus vaccine in intestinal mononuclear cells of gnotobiotic pigs transplanted with human gut microbiota. BMC Microbiol. (2016) 16:109. 10.1186/s12866-016-0727-2 27301272 PMC4908676

[33] Zhang L Li N des Robert C Fang M Liboni K McMahon R. *Lactobacillus rhamnosus* GG decreases Lipopolysaccharide-induced systemic inflammation in a gastrostomy-fed infant rat model. J Pediatr Gastroenterol Nutr. (2006) 42:545–52. 10.1097/01.mpg.0000221905.68781.4a 16707979

[34] Cicenia A Santangelo F Gambardella L Pallotta L Iebba V Scirocco A. Protective role of postbiotic mediators secreted by *Lactobacillus rhamnosus* GG versus Lipopolysaccharide-induced damage in human colonic smooth muscle cells. J Clin Gastroenterol. (2016) 50:S140–4. 10.1097/MCG.0000000000000681 27741159

[35] Fong FL Kirjavainen PV El-Nezami H. Immunomodulation of *Lactobacillus rhamnosus* GG (LGG)-derived soluble factors on antigen-presenting cells of healthy blood donors. Sci Rep. (2016) 6:22845. 10.1038/srep22845 26961406 PMC4785377

[36] Amdekar S Singh V. Studies on anti-inflammatory and analgesic properties of *Lactobacillus rhamnosus* in experimental animal models. J Complement Integr Med. (2016) 13:145–50. 10.1515/jcim-2015-0087 27078675

[37] Cai Z He W Zhuang FJ Chen Y. The role of high high-sensitivity C-reactive protein levels at admission on poor prognosis after acute ischemic stroke. Int J Neurosci. (2019) 129:423–9. 10.1080/00207454.2018.1538139 30332913

[38] Aoki-Yoshida A Saito S Tsuruta T Ohsumi A Tsunoda H Sonoyama K. Exosomes isolated from sera of mice fed Lactobacillus strains affect inflammatory cytokine production in macrophages *in vitro*. Biochem Biophys Res Commun. (2017) 489:248–54. 10.1016/j.bbrc.2017.05.152 28559134

[39] Isolauri E Kalliomäki M Laitinen K Salminen S. Modulation of the maturing gut barrier and microbiota: a novel target in allergic disease. Curr Pharm Des. (2008) 14:1368–75. 10.2174/138161208784480207 18537659

[40] Enticott PG Rinehart NJ Tonge BJ Bradshaw JL Fitzgerald PB. A preliminary transcranial magnetic stimulation study of cortical inhibition and excitability in high-functioning autism and asperger disorder. Dev Med Child Neurol. (2010) 52:e179–83. 10.3109/15622975.2015.1014411 20370810

[41] Tillisch K Labus J Kilpatrick L Jiang Z Stains J Ebrat B. Consumption of fermented milk product with probiotic modulates brain activity. Gastroenterology. (2013) 144:1394–401. 10.1053/j.gastro.2013.02.043 23474283 PMC3839572

[42] DuPaul GJ Power TJ Anastopoulos AD Reid R: ADHD Rating Scale-IV: Checklists, Norms, and Clinical Interpretation. New York, NY: Guilford (1998).

[43] Varni JW Seid M Rode CA. The PedsQL: measurement model for the pediatric quality of life inventory. Med Care. (1999) 37:126–39. 10.1097/00005650-199902000-00003 10024117

[44] Varni JW Seid M Kurtin PS. PedsQL 4.0. reliability and validity of the pediatric quality of life inventory version 4.0 generic core scales in healthy and patient populations. Med Care. (2001) 39:800–12. 10.1097/00005650-200108000-00006 11468499

[45] Varni JW. The PedsQL Measurement Model for the Pediatric Quality of Life Inventory (2020). Retrieved from https://www.pedsql.org/index.html 10.1097/00005650-199902000-00003 10024117

[46] Achenbach TM Rescorla LA Manual for the ASEBA Preschool Forms and Profiles. Burlington, VT: University of Vermont; Department of Psychiatry (2000).

[47] Achenbach TM Rescorla LA Manual for the ASEBA School-Age Forms and Profiles. Burlington, VT: University of Vermont; Research Center for Children Youth and Families (2001).

[48] Cohen J Statistical Power Analysis for the Behavioral Sciences. New York, NY: Routledge Academic (1988).

[49] Coghill D Hodgkins P. Health-related quality of life of children with attention-deficit/hyperactivity disorder versus children with diabetes and healthy controls. Eur Child Adolesc Psychiatry. (2016) 25:261–71. 10.1007/s00787-015-0728-y 26054300 PMC4769721

[50] Faber J Fonseca LM. How sample size influences research outcomes. Dental Press J Orthod. (2014) 19:27–9. 10.1590/2176-9451.19.4.027-029.ebo 25279518 PMC4296634

[51] Pahor D Systemic errors in clinical studies. Acta Medico Biotechnica. (2017) 10:7–11.

